# Compact bunker shielding assessment for 1.5 T MR-Linac

**DOI:** 10.1038/s41598-022-10498-0

**Published:** 2022-04-25

**Authors:** Jiwon Sung, Yeonho Choi, Jun Won Kim, Ik Jae Lee, Ho Lee

**Affiliations:** 1 Department of Radiation Oncology, Yonsei University College of Medicine, 50-1 Yonsei-ro, Seodaemun-gu, Seoul, 03722 South Korea; 2 Department of Radiation Oncology, Gangnam Severance Hospital, 211 Eonju-ro, Gangnam-gu, Seoul, 06273 South Korea

**Keywords:** Radiotherapy, Magnetic resonance imaging

## Abstract

This study evaluated the effect of the 1.5 T magnetic field of the magnetic resonance-guided linear accelerator (MR-Linac) on the radiation leakage doses penetrating the bunker radiation shielding wall. The evaluated 1.5 T MR-Linac Unity system has a bunker of the minimum recommended size. Unlike a conventional Linac, both primary beam transmission and secondary beam leakage were considered independently in the design and defined at the machine boundary away from the isocenter. Moreover, additional shielding was designed considering the numerous ducts between the treatment room and other rooms. The Linac shielding was evaluated by measuring the leakage doses at several locations. The intrinsic vibration and magnetic field were inspected at the proposed isocenter of the system. For verification, leakage doses were measured before and after applying the magnetic field. The intrinsic vibration and magnetic field readings were below the permitted limit. The leakage dose (0.05–12.2 µSv/week) also complied with internationally stipulated limits. The special shielding achieved a five-fold reduction in leakage dose. Applying the magnetic field increased the leakage dose by 0.12 to 4.56 µSv/week in several measurement points, although these values fall within experimental uncertainty. Thus, the effect of the magnetic field on the leakage dose could not be ascertained.

## Introduction

Magnetic resonance imaging (MRI) scanners are popular medical devices that direct radiofrequency (RF) pulses into the human body, resonating the hydrogen nuclei in tissue to emit signals. The signals from each tissue are converted into digital images by the scanner^1–3^. MRI provides higher contrast in the images of soft tissues such as fat, muscle, brain cells, ligaments, and tendons as compared with other diagnostic imaging technologies^4–6^. Moreover, MR images are acquired without any ionizing radiation, thus keeping patients safe from the harmful effects of radiation^7–9^.

The magnetic resonance-guided linear accelerator (MR-Linac), a linear accelerator and MRI scanner combination, is a recent medical development. It offers high-resolution real time MR imaging during treatment^10–13^. Similar to conventional MRI, MR images acquired via MR-Linac are affected by the electromagnetic noise of nearby geomagnetic fields and electronic devices.

Electromagnetic pulse frequencies used in MRI scanners range from 1 to 300 MHz, similar to the signals emitted from TVs, radios, and other household appliances^14^. If this electromagnetic noise is present in the vicinity of an MR-Linac, the MR-Linac receives them alongside the RF signals emitted from the body of the patient. This might reduce image quality or generate artifacts in the MR images. Conversely, the magnetic fields and RF signals emitted from the MR-Linac affect nearby electronic devices, such as medical devices. Therefore, MR-Linac requires not only a radiation shielding structure, but also RF shielding walls to both prevent surrounding electronic devices from contaminating the MR images and prevent MR signal interference with nearby medical devices^15^. The shielding walls of the MR-Linac should effectively shield against leakage doses through the numerous ducts for hose pipes and power, data, and control cables.

The 1.5 T MR-Linac Unity (Elekta AB, Stockholm, Sweden) introduced at our hospital is the first of its type in Korea. The magnetic field affects paths of the secondary electrons generated by radiation, which results in electron return effect (ERE) and electron stream effect (ESE)^16,17^. The Unity has a unique design in which the radiation beam passes through primary collimator, monitor chamber, multileaf collimator (MLC), V-shaped diaphragm, cryostat, gradient coil, and system body coil^18^. This structure results in increased radiation scattering compared to conventional LINAC. The shielding walls of the Unity bunker require additional shielding material to block electromagnetic noise from the outside. The constructed bunker was of the minimum size recommended by the vendor^19^. Unlike conventional Linacs, both primary beam transmission and secondary beam leakage were defined at the boundary of the machine and away from the isocenter. Therefore, the bunker wall was designed to be thick enough to block both primary beam transmission and secondary beam leakage. These physical phenomena and structural differences are in distinct contrast with a conventional Linac and are critical for determining the type and thickness of the shielding wall material, which has an impact on the cost of the 1.5 T MR-Linac. To the best of our knowledge, a study on the effect of magnetic fields on the leakage dose rate in compact bunkers housing a 1.5 T MR-Linac has not yet been reported. Therefore, we measured and verified the leakage doses at seven different locations, including at those around the ducts, met the criteria suggested by NCRP 151, and evaluated the magnetic field effect on leakage dose by comparing leakage dose measurements taken before and after applying the magnetic field.

## Results

**Figure 1:**
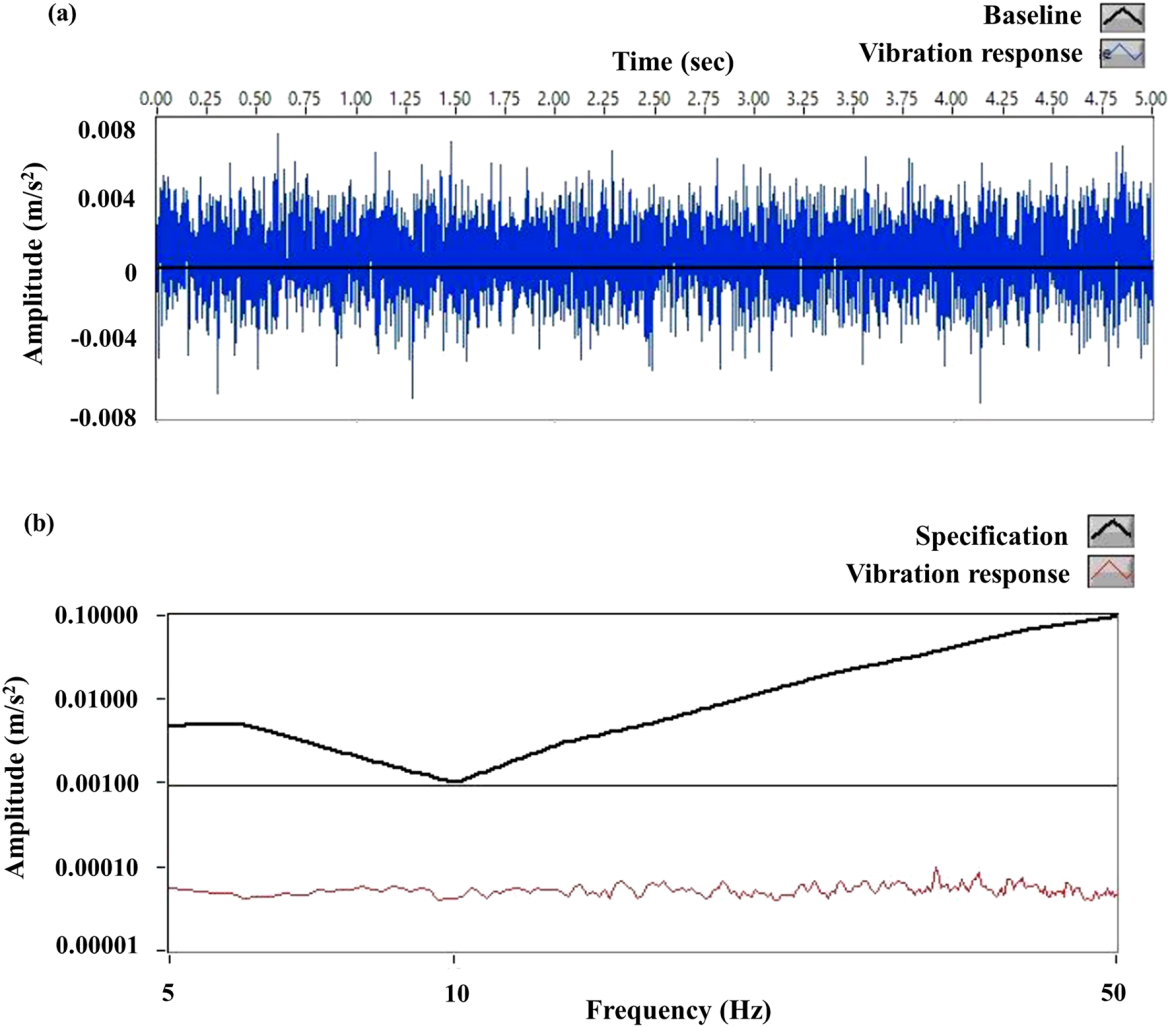
Magnitude of vibration according to (**a**) measurement time and (**b**) frequency.

Figure 1a shows the vibration magnitude measurements over 5 s; the vibration magnitudes were all similar. The results show that vibration levels are acceptable, as suggested by Philips, and that the Unity machine could be properly installed. Figure 1b shows that vibration was below the Philips Marlin 1.5 T specification for the frequency range of 1 to 50 Hz.

**Figure 2:**
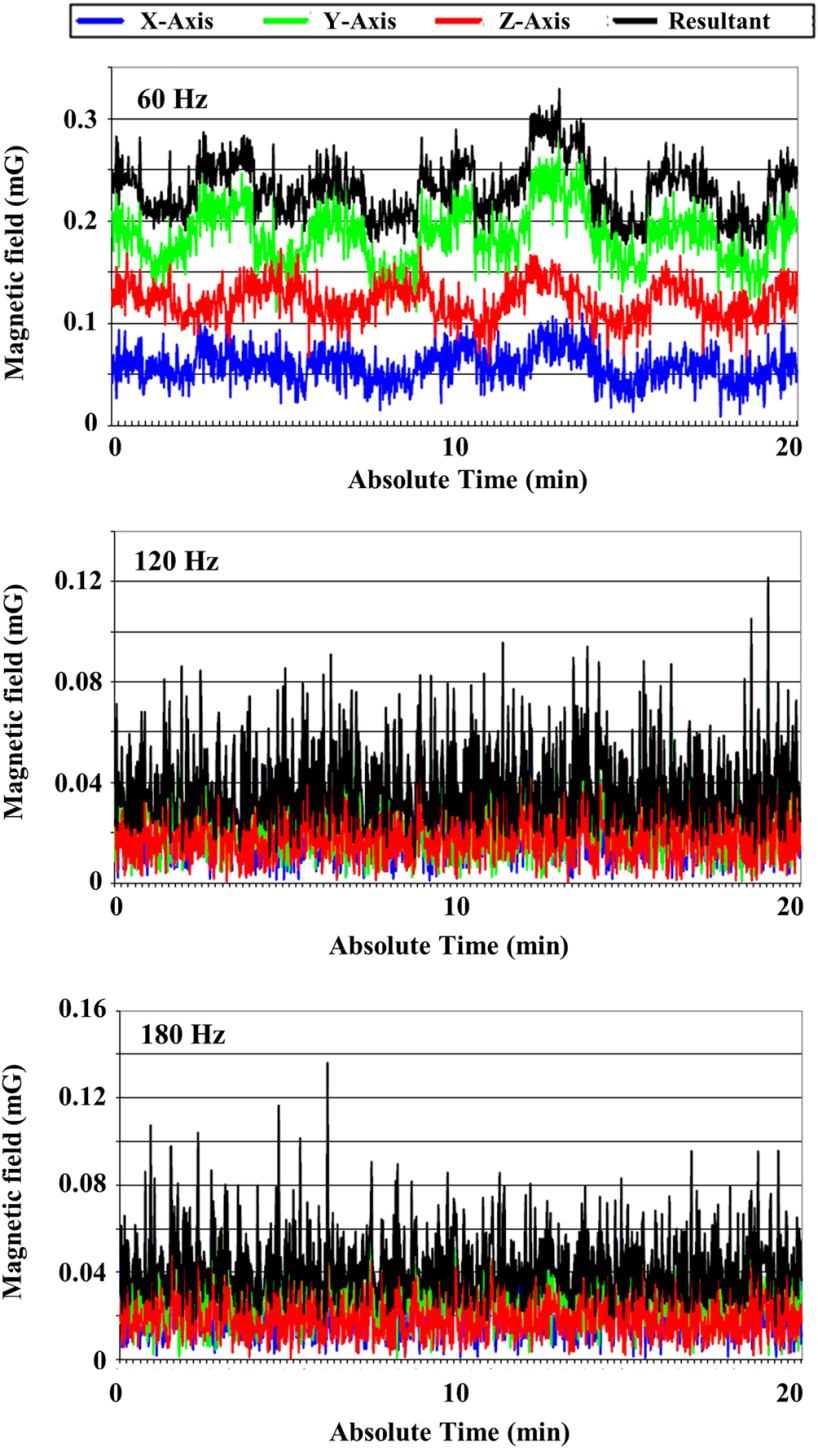
AC magnetic field measurement over 20 min.

**Figure 3:**
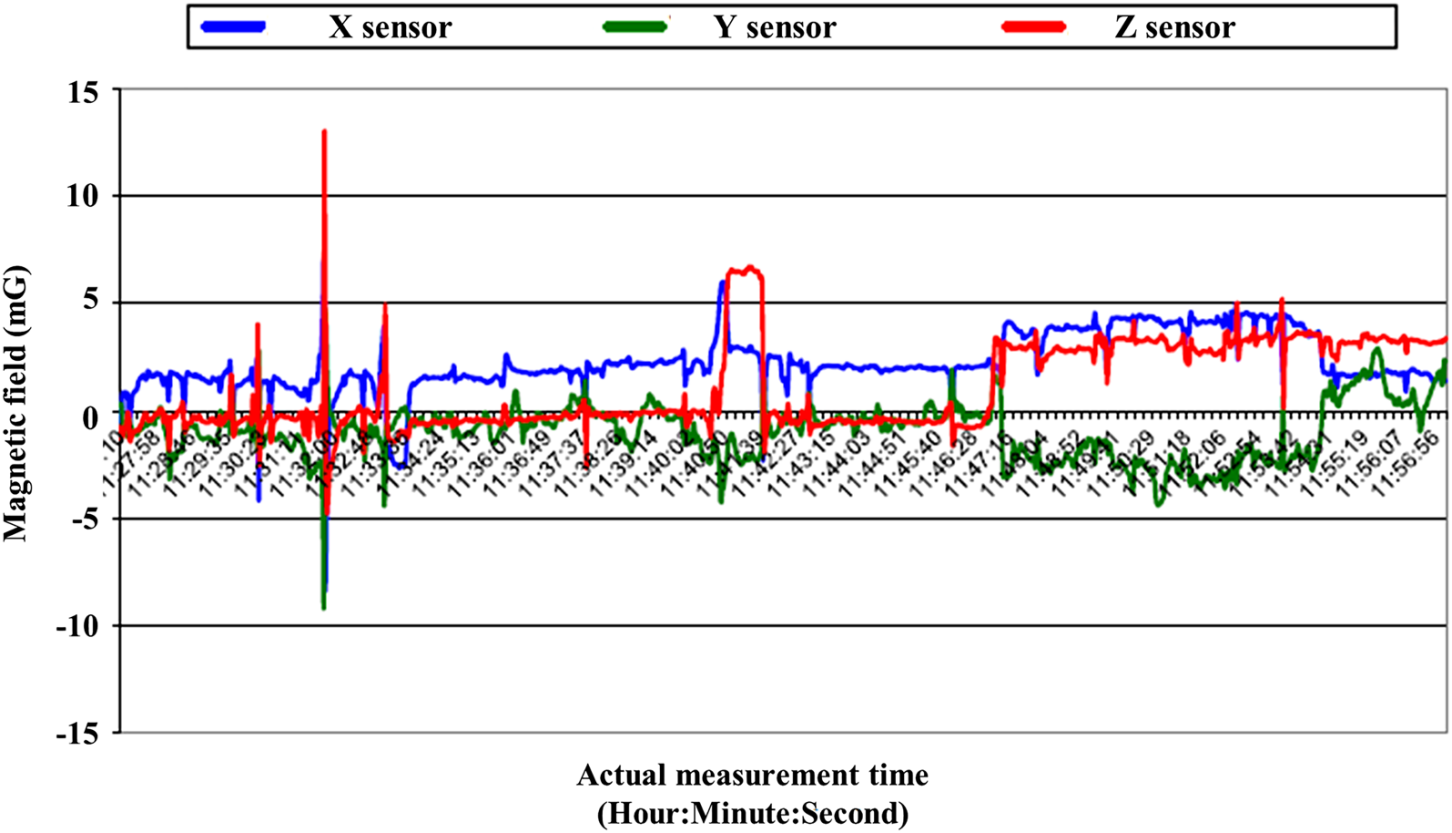
DC magnetic field measurement for 30 min.

Alternating current (AC) magnetic field disturbances were below 1.0 mG in all axes (Fig. 2). Direct current (DC) magnetic field fluctuations were below 8.41 mG, 9.23 mG, and 13.05 mG in the X, Y, and Z-axis, respectively (Fig. 3).

Table 1 shows the leakage dose values measured before and after applying MR. The total leakage doses at all measurement points were below the NCRP standard criteria of 20 or 100 μSv/week. The average total leakage doses due to the primary beam were measured as 0.115 and 0.063 μSv/week before and after the static magnetic field of 1.5 T was turned on, respectively. The average total leakage dose due to the secondary beam was measured as 4.437 and 4.794 μSv/week before and after the static magnetic field of 1.5 T was turned on, respectively. These results show that, regardless of whether the MR was turned on, the leakage doses due to the secondary beam were larger than those due to the primary beam.

**Table 1:** Comparison between leakage doses before and after applying 1.5 T MR.

Type	#	Photon		Neutron		Sum (Photon + Neutron)		Criteria (μSv/week)
		Before MR (μSv/week)	After MR (μSv/week)	Before MR (μSv/week)	After MR (μSv/week)	Before MR (μSv/week)	After MR (μSv/week)	
*PR	①	0.116	0.052	0.000	0.000	0.116	0.052	100
	⑥	0.115	0.075	0.000	0.000	0.115	0.075	20
*SR	②	11.940	8.298	0.290	0.000	12.230	8.298	100
	③	0.427	0.544	0.000	0.000	0.427	0.544	100
	④	4.958	9.512	0.000	0.000	4.958	9.512	20
	⑤	1.579	1.214	0.000	0.000	1.579	1.214	20
	⑦	2.530	1.963	0.460	2.440	2.990	4.402	20

The numbers indicate measurement positions shown in Fig. 6.

*PR* primary radiation, *SR* secondary radiation.

With regard to the leakage doses measured before the MR was applied, the maximum average leakage dose due to the secondary beam was 12.230 μSv/week in the control room. The next highest leakage dose of 4.958 μSv/week due to the secondary beam was observed in the waiting room; the remaining measuring points showed similar levels of leakage. This is attributed to the reflection of photon leakage within the bunker. The highest value of photon leakage dose due to the secondary beam was 11.940 μSv/week in the control room. The next highest leakage dose, 4.958 μSv/week due to the secondary beam, was in the waiting room, with the remaining doses exhibiting similar levels. The neutron leakage doses, however, were negligible at all measurement points.

As regards the leakage doses measured after the MR was applied, the maximum average leakage doses due to the secondary beam were 9.512 μSv/week in the waiting room and 8.298 μSv/week in the control room. This is the result of photon leakage reflection, and shows little change from the dose without MR. The photon leakage doses due to the secondary beam were also measured to be as high as 9.512 μSv/week in the waiting room and 8.298 μSv/week in the control room, with the remaining doses measured to have similar levels. The measured values of the neutron leakage doses were negligible. The increase in leakage dose with applying MR ranged from 0.12 μSv/week in the treatment door to 4.55 μSv/week in the waiting room.

**Figure 4:**
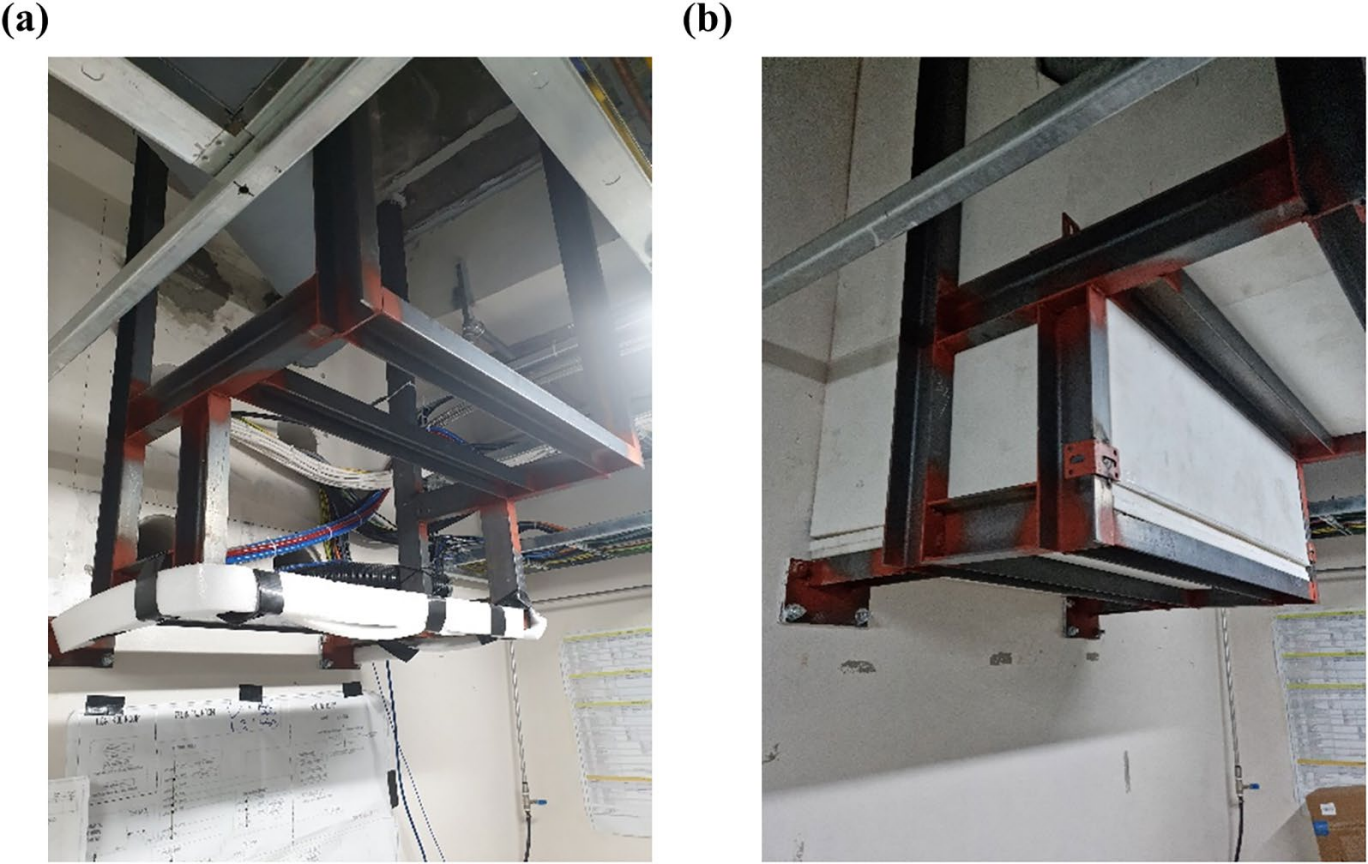
Comparison (**a**) before and (**b**) after the special shielding design to reduce leakage dose around the ducts.

Figure 4 shows image of the duct before and after shielding. Unlike the existing method, the duct was shielded by attaching a shielding material from the outside. Although the maximum leakage dose was measured around the ducts, leakage was reduced by a factor of 5 by shielding the duct.

## Discussion

The unique design of Unity demonstrates characteristics that differ from those of the conventional Linac. Interaction between the primary beam and MR-related devices such as the cryostat and MR coils generates additional scattered radiation. In addition, secondary electrons generated via the interaction between photons and air, are influenced by the static magnetic field and exhibit spiral movement. Owing to the movement of these electrons, the scattered beam can be formed wider than the expected area compared to the conventional Linac^20^. In particular, even if the magnetic field strength is small, it can occur from the leakage radiation passing through the duct. Many studies related to this have been published^21–24^, but studies on the effect of the MR leakage dose s have not been reported yet. In addition, our bunker was designed to be the smallest within the range suggested by the manufacturer due to the spatial limitations. Therefore, this study estimated whether 1.5 T magnetic field affects the leakage doses passing through the radiation shielding wall.

Before the installation of MR-Linac, the magnitude of vibration and magnetic fields was measured at the installation location to obtain baseline measurements. Vibration responses were below the magnet specification limit (Philips Marlin 1.5 T specification for the frequency range of 1 to 100 Hz) during the time of the examination. Specific sources for the measured vibration responses could not be identified during the examination.

DC magnetic field fluctuations were below 13.05 mG during the examination. The main source for DC magnetic field disturbances was from above the main entrance driveway, approximately 3 m away from the proposed isocenter. A proven solution to compensate for DC magnetic field disturbances created by moving metal (elevators, trains, subways, vehicles etc.) would be the ETS-Lindgren magnetic active compensation system (MACS). This system can be installed during the construction of the RF shielded enclosure; with this system installed, the DC magnetic field disturbances will normally be reduced to 1 mG pk-pk or less at the MRI isocenter. All AC magnetic field disturbances were below 1.0 mG. After a visual inspection of the proposed MRI area, no AC magnetic field sources were located that had measureable disturbances greater than 1 mG within 15.24 m of the measurement location.

The results in Table 1 confirmed that leakage doses in all measurement positions satisfied the criteria suggested by NCRP 151. Instantaneous leakage dose rates below 20 μSv/hr were measured, a level acceptable to the Korea Institute of Nuclear Safety.

To evaluate whether the magnetic field generated in Unity affects the leakage doses, we compared the leakage dose before and after applying MR in the shielding walls and in the vicinity of the duct. The results revealed that the leakage doses before and after applying MR are similar. Three explanations exist for these results. First, the instantaneous leakage dose rate itself is very low. Second, the instantaneous leakage dose rate changes in real time, and the measurement location selection changes the highest measured value. Third, the survey meter itself contains an uncertainty range. It can be said that the degree of influence of the leakage dose resulting from the application of MR is insignificant because it is within the uncertainty range. Comparing the leakage dose around the duct before and after additional shielding indicated that the instantaneous dose rates are similar, with an average of 1.362 μSv/hr before applying MR and 1.64 μSv/hr during applying MR. Therefore, the effect of MR on the leakage dose can be concluded to be negligible.

## Conclusion

A 1.5 T MR-Linac system was installed at our hospital for the first time in Korea. The bunker was constructed with the minimum bunker size recommended by the vendor. This study evaluated the 1.5 T magnetic field produced by the MR-Linac to determine leakage dose escaping the shielding walls in our bunker. The results did not show any significant difference between leakage doses before and after applying 1.5 T MR. Further, all measured leakage doses satisfy the dose limits proposed by NCRP 151.

## Materials and methods

### Structural characteristics and room layout

The Unity, an MR-Linac model, was approved for clinical use by the U.S. Food and Drug Administration (FDA) for patient treatments in 2018^25^. The Unity combines a 7 MV standing‐wave Linac and a 1.5 T Philips big‐bore MRI (Philips Healthcare, Amsterdam Netherlands). The system has a source axis distance (SAD) of 143.5 cm and a maximum field size of 57.4 × 22 cm with field defining diaphragms in the cross-plane and 160 MLC leaves in the in-plane direction.

**Figure 5:**
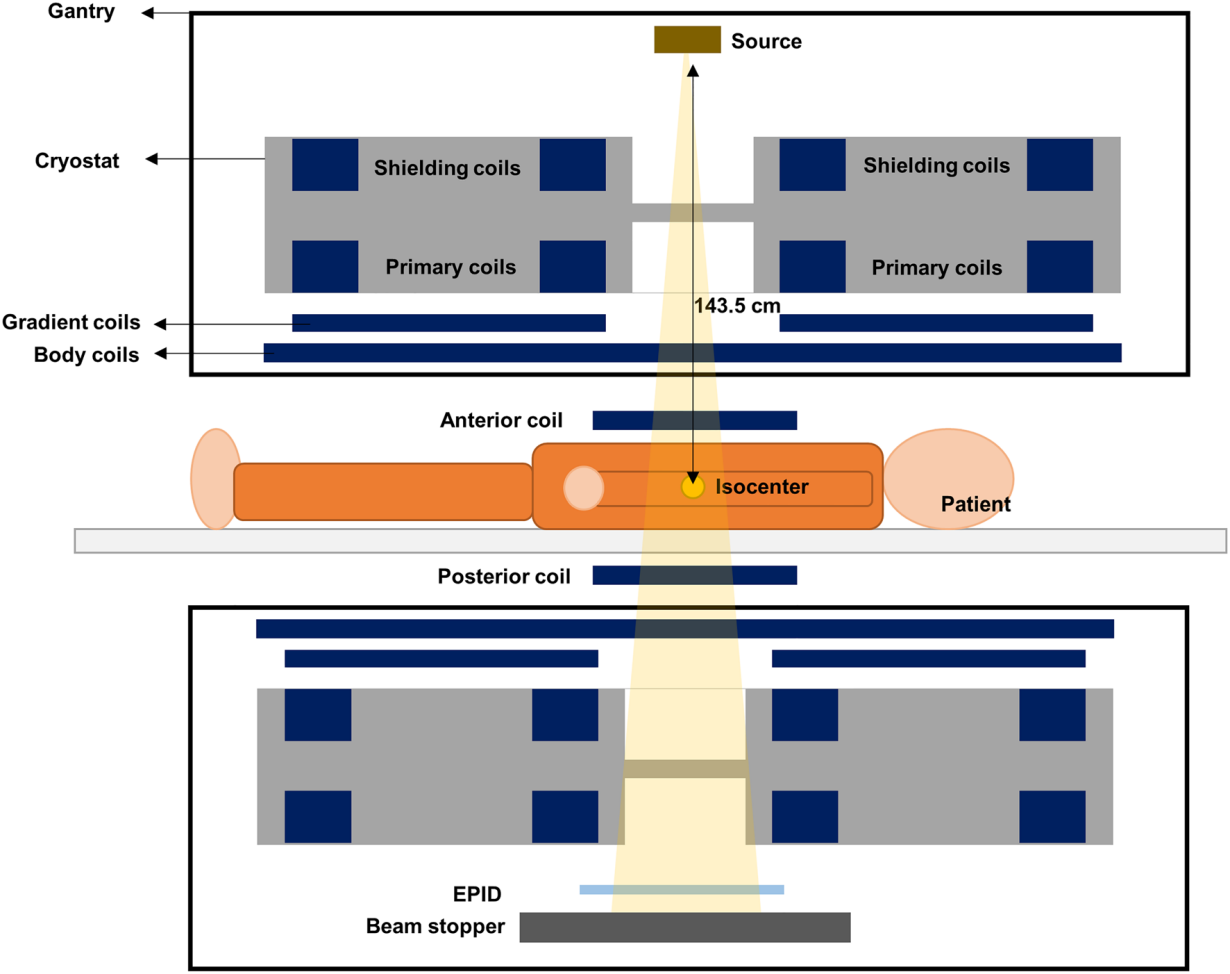
Unity structure.

The Unity system is a bore-type machine in which a linear accelerator rotates around the MRI system. The nonstandard SAD results in different beam characteristics, such as the profile shape and percentage dose depth compared to the conventional 100 cm SAD. The beam path of the MR-Linac includes the MR cryostat, gradient coil support structure, quadrature body coil (QBC), anterior and posterior receiver coils, and the patient support system (Fig. 5).

**Figure 6:**
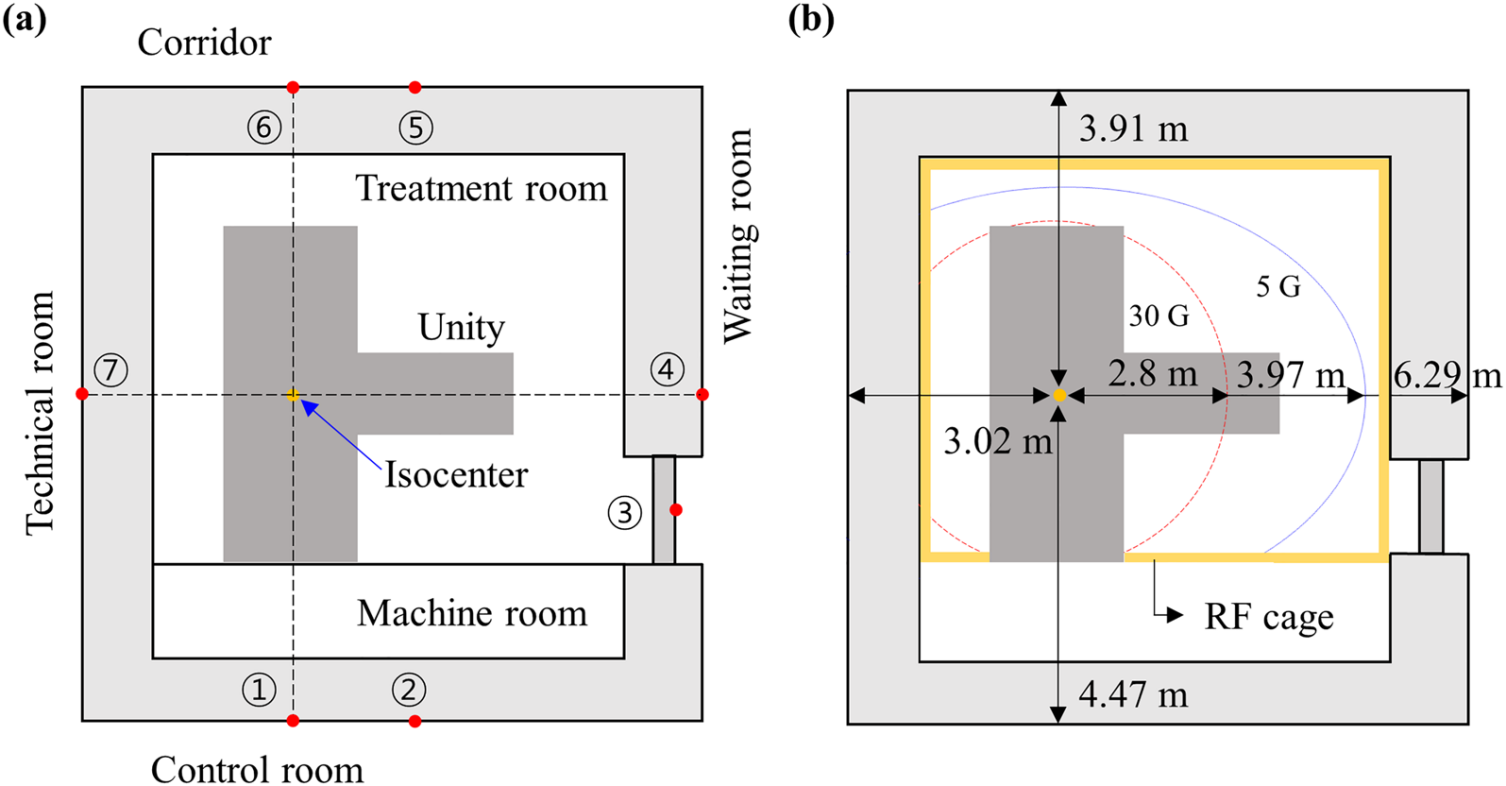
Unity area layout including (**a**) measurement locations (red dots) and (**b**) distances between isocenter and shielding walls.

The Unity space is divided into four areas: treatment, machine, control, and technical room, as shown in Fig. 6. The treatment room contains the treatment delivery system; that is, the primary source of radiation and the field generator unit (FGU). At the bottom, a circle denotes the 30 G magnetic field cutoff where the risk of ferromagnetic objects becoming projectiles is elevated. The 5 G cutoff is contained within the outer edges of the walls of the treatment and machine rooms. The treatment room is shielded by a radiofrequency (RF) cage composed of copper to prevent extraneous electromagnetic noise generated by the MR from interfering in nearby medical devices. The machine room is located within the treatment room but is outside the RF cage. This room allows access to the gantry components and RF cage filter panel; it contains the service crane, raised access floor, and heat exchanger. Moreover, there is the area where cables and services connect to the RF cage. The control room is intended for the radiation therapist or other radiation staff who operate the MR-Linac to treat patients and observe patient movement during treatment. This room contains the Unity operator’s console, workstations, and CCTV monitor. The technical room contains the treatment delivery system cabinets and MR cabinets required for the operation of Unity.

### Shielding design

The Unity room established in our hospital is smaller in size than conventional rooms because it was designed for an earlier treatment machine that has been decommissioned. Due to spatial limitations, the maze corridor recommended by the supplier to reduce leakage doses was not included in our Unity room structure. Based on the criteria suggested in NCRP 151^26^, the control room and entrance to the treatment room were designated as controlled areas, whereas the others were designated as uncontrolled areas. According to this classification, the shielding wall was designed such that the leakage dose in the controlled areas and uncontrolled areas do not exceed 100 and 20 μSv/week, respectively.

Two types of radiation barriers are typically considered in this case, namely primary and secondary. The primary barriers are exposed to direct photon radiation from the target or source, whereas the secondary barrier receives scattered radiation resulting from the primary beam hitting the surfaces of the treatment room, in addition to radiation transmitted through the accelerator head.

The parameters of the shielding wall were calculated using the formulas presented in Table 2.

**Table 2:** Parameter formulas for the primary and secondary beam.

	Primary barrier	Secondary barrier
B	$\frac{{Pd^{2} }}{{W_{p} UT}}$	$\frac{{Pd^{2} }}{{10^{ - 3} W_{s} UT}}$
N	$- \log B_{p}$	$- \log B_{s}$
T	$n_{p} \times TVL_{p}$	$n_{s} \times TVL_{s}$

In the parameter formulas, P is the shielding design goal beyond the barrier; d is the distance from the x-ray source or isocenter to the protected point; and W is the workload, specified as the absorbed dose from photons delivered to the isocenter in a week. The secondary barrier workload W_s_ was calculated via intensity modulated radiotherapy (IMRT) factor. The use factor U is the fraction of a primary-beam workload that is directed toward a given primary barrier; the occupancy factor T is the fraction of the workweek for which a person is present beyond the barrier; TVL is the tenth value layer determined by the radiation energy and shielding material, and t is the barrier thickness. According to the suggestions of NCRP 151, the values of P, U, and T depend on shielding location. Therefore, these were calculated according to the dimensions of the bunker. Moreover, although Unity uses 7 MV energy, the TVL values were selected for an energy value of 10 MV to further reduce the leakage doses.

Typically, neutrons are not produced by low-energy photons (≤ 10 MV) incident on the various materials of target, collimators, and other shielding components^27^. However, Unity has been shown to produce small numbers of photo-neutrons, with a fluence of less than 3 × 10^4^ cm^−2^ Gy^−1^ at the isocenter and less than 9 × 10^3^ cm^−2^ Gy^−1^ at a distance of 90 cm from the isocenter along the MR bore^19^. This neutron fluence can cause neutron leakage out of the bunker, especially if a maze is not present^19^.

As power, data, control, and hose piping require the construction of ducts, additional shielding is required. The ducts are first shielded with the wall thickness calculated using the formula for the secondary barrier. Additional shielding is then implemented where the leakage dose from the duct is higher than the leakage dose limit. The lead thickness used for the duct shielding is 30 mm, while the borated polyethylene (BPE) thickness is approximately 40 mm. The materials and thickness of the shielding walls for each location are shown in Table 3.

**Table 3:** Parameters and information for shielding walls according to each location.

Type	Figure 2a	P (μSv/week)	d (m)	W (Gy/week)	U	T	Shielding thickness			
							Concrete (cm)	Lead (cm)	BPE(cm)	Equiv. concrete (cm)
PR	①	100	4.47	1700	0.22	0.2	79.0	15.0	0	180.8
	⑥	20	3.91	1700	0.22	0.2	42.3	21.0	0	184.8
SR	②	100	4.52	8500	1	0.2	86.1	9.1	0	141.4
	③	100	6.27	8500	1	0.125	0.0	22.0	10	133.7
	④	20	6.29	8500	1	1	120.0	5.0	0	150.4
	⑤	20	3.99	8500	1	0.2	45.2	19.3	0	162.5
	⑦	20	3.02	8500	1	0.05	50.0	13.0	0	129.0

The numbers indicate measurement location shown in Fig. 6.

*PR* primary radiation,*SR secondary radiation.

### Preliminary examination for the intrinsic vibration and magnetic fields

**Figure 7:**
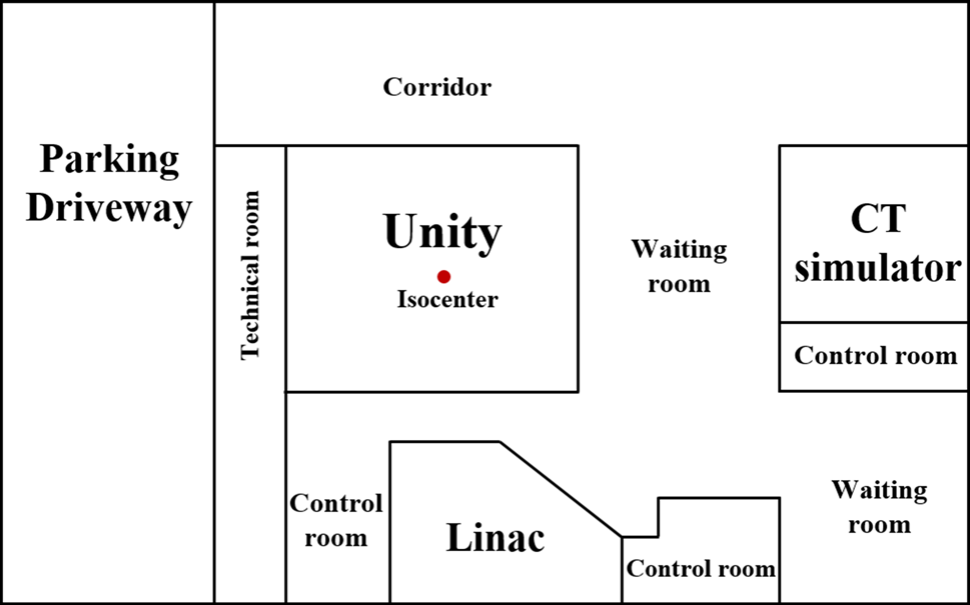
Location of vibration and magnetic field examination (red point).

Prior to the installation of MR-Linac, the vibrational magnitude and magnetic fields were measured at the installation location to determine the baseline parameters. Vibrational analysis is commonly conducted both on the vibrational time-domain and frequency-domain signals; frequency domain data are obtained by applying a Fourier transform to the time-domain waveform. The vibration data were collected using one PCB Piezotronic seismic accelerometer positioned at the proposed isocenter location, as shown in Fig. 7. Data were collected over a 60-min interval using a 24-bit data acquisition system with a sensitivity of 10 V/g.

The AC and DC magnetic field analyses were performed by positioning a magnetometer at the proposed location of the isocenter, as shown in Fig. 7. Both analyses were conducted over a 30-min interval with the magnetometer set at a height of 1 m. The AC measurement was set up to measure ambient AC magnetic fields at the frequencies of 60, 120, and 180 Hz for the X, Y and Z-axes, as well as the vector resultant. All AC and DC magnetic field measurements were conducted using a 24-bit data acquisition system and a magnetometer with a range of 10 G. All data were acquired with 1000 linear samples at a scan rate of 1000 samples per second.

### Evaluation of MR effect for leakage doses

The effect of the 1.5 T magnetic field on the leakage dose was evaluated by comparing the leakage doses measured before and after the 1.5 T magnetic field was applied. We measured leakage dose using a gamma survey meter (S. E. International, Inc., TN) and neutron meter (LUDLUM Measurement, Inc., TX). The gamma survey meter and neutron survey meter were calibrated in January 2021 and May 2021 by accredited calibration laboratories, respectively. The radiation source used for gamma survey meter calibration was a Cs-137 source; the average calibration factor was 1.132 × 10^−2^ Sv/R with a measurement uncertainty of 6.6%. The radiation source used to calibrate the neutron survey meter was a Cf-252 source, with a calibration factor of 4.5 μSv/hr and measurement uncertainty of 8.5%.

The measurement locations for the leakage dose passing through the shielding wall are shown in Fig. 6a. In practice, as a conservative approach, we measured several points around each measurement location and recorded the highest reading. The number of experiments executed for each measurement point was at least two. Because the measured values (instantaneous dose rate) change in real time, measurements were taken for approximately 20 to 30 s to ensure that the most stable value was recorded at each position. Two measuring points were located in each of the control room and corridor. The first was located at a primary radiation leak and the other at a secondary radiation leak. The remaining rooms had one measurement point each at secondary radiation leaks. The irradiation conditions (maximum field size, dose rate, and gantry angle) vary depending on the measurement point. When measured from the point in the waiting and technical rooms, the gantry moves at 0°; when measured from the point in the control room and corridor, the gantry moves at 90° and 270°, respectively.

## Data Availability

All data generated or analyzed during this study are included in the article.
